# Impact of Wheat Crop Management on Co-occurrence of Group B Trichothecenes: Crop Practices Influencing DON and Derivatives in Wheat

**DOI:** 10.1021/acs.jafc.6c00387

**Published:** 2026-03-26

**Authors:** Agápto João Paulo, Júnior Waldir Cintra de Jesus, Afférri Flávio Sérgio, Carmassi Alberto Luciano, Lemos Andressa Cunha, Badiale-Furlong Eliana, Scaglioni Priscila Tessmer

**Affiliations:** † Campus Lagoa do Sina, Centro de Ciências da Natureza, Universidade Federal de São Carlos, Buri, São Paulo State 18245-970, Brazil; ‡ 67820 Universidade Federal do Rio Grande, Escola de Química e Alimentos—Campus Carreiros, Avenida Italia km8, Rio Grande, Rio Grande do Sul State 96203-900, Brazil

**Keywords:** fungicides, potassium silicate, 3-ADON, 15-ADON, DON-3G, NIV

## Abstract

Profiles of deoxynivalenol (DON), 3-acetyl-DON (3-ADON), 15-acetyl-DON (15-ADON), DON-3-glucoside (DON-3G), and nivalenol (NIV) were evaluated in wheat grown in an experimental field under different agronomic conditions regarding their roles in co-contamination with group B trichothecenes (TCTBs). For three years in southwestern Brazil, a susceptible and moderately resistant cultivar was cultivated in irrigated and rainfed systems. A randomized complete block design was carried out to evaluate the effects of the treatments: T1 (fungicide + potassium silicate (KS)), T2 (fungicide), T3 (KS), and T4 (control) on the TCTBs profile that was determined by the validated QuECHERS-HPLC-PAD method. Co-contamination occurred in 79.2% of the samples and the highest level for 15-ADON (1640 μg/kg). In 20 samples, the sum of TCTBs was above the maximum tolerable limit (MTL) for DON (1000 μg/kg). Samples from T1 and T3 had lower contamination levels. It is advisable to consider KS fertilization and include DON forms in MTL to reduce contamination risk.

## Graphical abstract

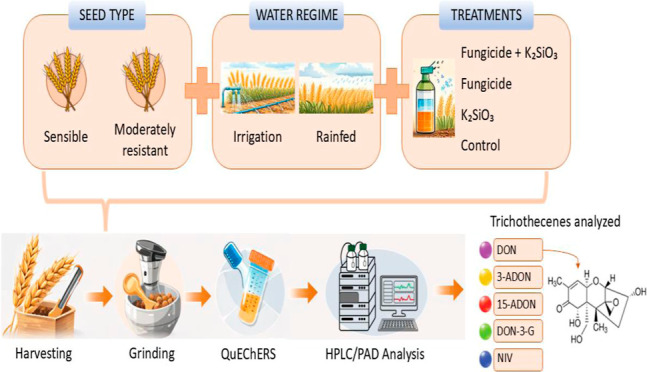

## 1 Introduction

Wheat (*Triticum aestivum L.*) represents 30% of the grains used as sources of calories and protein for humans and animals. Wheat crop contamination by fungus and production of mycotoxins may be favored by grain composition, crop management, and the climate scenario.^1,2^ Since wheat-based products are widely consumed in the global diet, therefore, biological or chemical contamination of the grain represents a challenge to ensuring food safety^3,4^ (see Figure 1).

**Figure 1:**
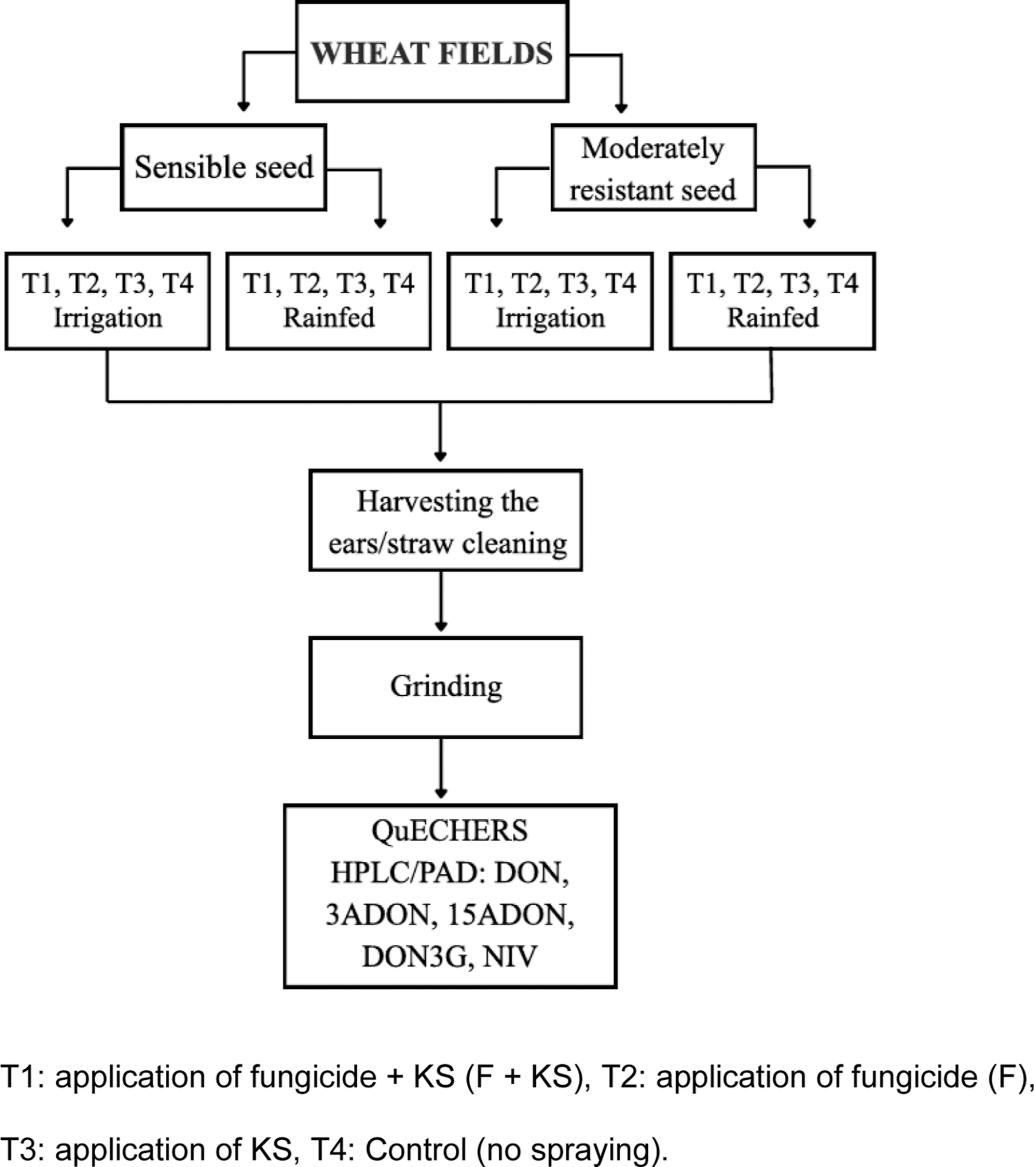
Experimental diagram.

The *Fusarium graminearum* species complex (FGSC), which includes *Fusarium acacia-mearnsii*, *Fusarium aethiopicum*, *Fusarium asiaticum*, *Fusarium austroamericanum*, *Fusarium brasilicum*, *F. graminearum, Fusarium meridionale,* and *Fusarium vorosii* and genera *Myrothecium*, *Cephalosporium*, *Verticimospoiun,* and *Stachbotrys,* ^3,4^ infects wheat plants in the stages of anthesis and grain filling. It impacts on productivity, quality, and safety of wheat grains.^2,4^

In the TCTBs group, deoxynivalenol (DON) stands out not due to its frequent occurrence in grains produced worldwide and its gastrointestinal, immunotoxicity, hematologic, and neurotoxic effects.^5^ It is related to activities at the molecular level and inhibits different stages of protein synthesis.^6^ To mitigate exposure to damage caused by TCTBs, sanitary authorities in several countries have established maximum tolerable limits (MTL) that range from 250 to 2000 μg/kg for DON in wheat products.^2,7,8^

Derivatives of DON (acetylated and glycosylate forms) and NIV have drawn attention^5,9,10^ because their detection in several cereal has increased, known toxic potential, evidence of interconversion to DON in the matrix and in the digestion system.^5^ ^,^ ^7^ ^,^ ^8^ In food matrices, production of DON is a response to genetic characteristics of the fungus species, defense mechanisms of the host,^11^ crop management,^12–15^ type of fungicides,^16–19^ climate parameters,^13,20^ and storage and food processing,^7,21^ highlighting that the pathway of DON and its derivatives synthesis is similar and may also be affected by these multifactorial parameters.^2,8^

To prevent wheat contamination with the FGSC followed by contamination with DON, some tools have been used, i.e., selection of resistant varieties,^11^ soil fertilization,^14^ induction of defense mechanisms of the host, irrigation systems, and fungicide application,.^22–24^ However, there is scarce information about the effect of these tools for avoiding co-contamination with DON and its derivatives.

Simultaneously validated methods for detection of DON chemical forms comprise the first stage to improve the understanding of the effects of agronomic variables on TCTBs production by the FGSC.^4,6,8^ This knowledge is fundamental to propose predictive models of multiple wheat contamination which can help estimate the true risk of TCTBs occurrence and promote the development of strategies to mitigate the impact on human health.^8,23^

Intending to fill the gap regarding the role of agronomic variables on co-occurrence of TCTBs, a three year study was carried out in an experimental field located at the subtropical region of the state of São Paulo, Brazil. The study aimed to evaluate the profiles of DON, 3- 3-ADON, 15-ADON, DON-3G, and NIV in wheat grown in an experimental field under different agronomic conditions, i.e., seed susceptibility, irrigation system, fungicide, and potassium silicate (KS) fertilization. The results should help to prevent wheat contamination with TCTBs and to review the MTL for DON.

## 2 Materials and Methods

### 2.1 Materials

Wheat seeds planted on experimental fields belong to moderately resistant (MR) and susceptible (S) cultivars registered in the Registro Nacional de Cultivares (RNC) *n* ^o^ = 32218 and *n* ^o^ = 31487, respectively.

Standards of mycotoxins DON, 3-ADON, 15-ADON, DON-3-G, and NIV were purchased from Sigma-Aldrich. Solvents were of HPLC grade, while salt and adsorbent were of analytical grade.

Fungicide (Nativo) trifloxystrobin (100 g/L) + tebuconazole (200 g/L) at the dose of 0.75 L/ha was complemented with methylated soybean oil adjuvant at the dose of 0.25% of the spray volume.

KS was composed of silicon (Si) 12% and potassium (K_2_0) 12% at the density of 1.38 g/cm^3^ and pH equal to 10.96 at the dose of 1.5 L/ha^1^.

### 2.2 Experimental Section

Field experiments were carried out on the Lagoa do Sino campus at the Federal University of São Carlos (latitude 23°36′4.65″S; longitude 48°31′25.53″W; and altitude 637 m) in the southwestern São Paulo state. The experimental area had 0.5 irrigated hectares and 0.5 rainfed ones. The climate in the region is classified into “Cwa” by Koppen. Winter is dry, the temperature is below 18 °C, summer is hot (above 22 °C), and the rainfall index is 1300 mm/year. The soil is classified into typical dystrophic red latosol, moderate A, clayey, or very clayey texture, gently undulating relief.^25^

The plotted area was 15 m^2^ (2.5 m × 6 m), spacing between rows was 0.17 m, and seeding density ranged from 300 to 350 plants/m^2^. To avoid interference with treatments, three rows on both sides of the area were discarded.

Five irrigation events were carried out by 12 mm blades for 30 min in the central pivot area from sowing to harvest. In the period between extrusion of anthers and the end of the anthesis, three irrigation events were carried out. Irrigation was always conducted at 9:30 am.

### 2.3 Experimental Design of Wheat Cultivation

Eight experiments were carried out involving combinations of years (sowing on May 11, 2021; on May 16, 2022; and May eighth, 2023), cultivation systems (irrigated and rainfed), and cultivars (MR and S). A randomized complete block design was used for evaluating the effects of the following treatments:

T1—application of fungicide + KS (F + KS), T2—application of fungicide (F), T3—application of KS, and T4—Control (no spraying).

Regarding fertilization, 300 k/ha 08-28-16 (N–P–K) and 200 k/ha 45% urea (N) (which is routinely used on the farm) were applied as the top dressing 38 days after sowing in all treatments.

Zadoks phenological scale was applied to standardize application of fungicides when wheat plants were more vulnerable to the FGSC. The first application occurred at stage 60 (preanthesis), the second, at stage 65 (anthesis) and the last, at stage 69 (postanthesis) on Zadoks scale.^26^

### 2.4 Sampling

Samples were randomly collected at stage 91 of Zadoks phenological scale.^26^ Ears were threshed, and each subsample, with approximately 100 g of grain weight, was dried and stored in kraft paper bags until the analysis of mycotoxins. Analytical samples were ground in a knife mill and sieved to a particle size of 32 mesh.

### 2.5 Determination of DON, Derivatives and NIV

Type B trichothecenes, specifically DON, its acetylated derivatives 3-ADON, 15A-DON, DON-3G and NIV were determined by the QuEChERS method proposed by Cerqueira et al.,^27^ adapted and validated to wheat grains. Performance indicators of mycotoxins, quantification limits of the instrument, quantification limits of the method (based on the dilution of the samples), linearity, recovery at three levels, precision, and the matrix effect were evaluated.

The validated protocol consisted of: weighing 3.5 g of sample in a 50 mL polypropylene tube. Ten mL of ultrapure water was added and vortexed for 1 min. Then, 10 mL of hexane was added to the mixture, which was homogenized in a vortex for 1 min and in an orbital shaker at 200 rpm for 15 min. The crude extract was centrifuged at 3220*g* for 10 min; the upper phase, which contained hexane and fat, was discarded. In the extraction and cleaning stage, 10 mL of acetonitrile was added and vortexed for 1 min, followed by the addition of 4 g of anhydrous magnesium sulfate and 1 g of anhydrous sodium chloride, vortexed for 1 min, and centrifuged at 3220*g* for 10 min. From the supernatant, 2 mL was transferred to 15 mL polypropylene tubes containing 300 mg of anhydrous magnesium sulfate and 150 mg of neutral alumina, vortexed for 1 min, and centrifuged at 3220*g* for 10 min. From the cleaning extract (supernatant), 1 mL was transferred to a vial and dried at 60 °C in a sand bath. The dried extract was resuspended in 1 mL of acetonitrile: water solution (50:50, v/v), vortexed for 1 min, and transferred to the vial for automatic injection into HPLC.

An HPLC system (Shimadzu, Tokyo, Japan) with a photodiode array detector (PAD) was used for determining mycotoxins. Chromatographic separation was performed with acetonitrile: ultrapure water (50:50, v/v) at the flow rate of 0.5 mL/min, oven temperature of 40 °C, elution in isocratic mode for 10 min, injection volume of 20 μL and maximum absorption wavelength of 220 nm. Mycotoxin identification was based on retention times and UV spectra of samples by comparison with the standard solution.^7^ ^,^ ^27^ Mycotoxins were confirmed by cochromatography and by comparison to UV spectra of the standard solution.

### 2.6 Data Analysis

Results of the individual analytical determination of DON, NIV, 3-ADON, 15-ADON and D3G, and the sum of detected levels in each sample, were evaluated by the analysis of variance (ANOVA) carried out by the computational program Statistica (STATSOFT 7). Normal distribution of residues was verified by the Kolmogorov–Smirnov test while homogeneity of the variance was evaluated by the Levene test. To conduct multiple comparisons, means of treatments were used by the Fisher F test (*p* < 0,10). Whenever necessary, transformation *T* = ln (*x* + 1) was used for normalizing data.

## 3 Results and Discussion

### 3.1 Agronomic Parameters

Climate characteristics in the experimental field were the ones expected in subtropical regions, i.e., dry and cold winter and hot spring (25–30 °C). Rainfall and relative humidity were lower in July and August (preanthesis and postanthesis). Variables related to the climate, i.e., temperature, relative humidity, and rainfall, in the critical period for mycotoxicological contamination in fields are in the Supporting Information. These variables may affect fungal pathways in grains and food matrices and change the profiles of trichothecenes production.^8,28,29^

Agronomic variables chosen for the experiments were the ones related to biotic (seed susceptibility) and abiotic factors (irrigation system, fungicide, and KS fertilization), which had previously been related to contamination with the FGSC and production of trichothecenes (mainly DON).^30^ Crop treatment with fungicide has been used worldwide to avoid fungal infection while soil fertilization with KS induces natural defense of the host.^31,32^ However, there is evidence that some fungicides may avoid fungal disease by stressing fungi before their inactivation, thus, promoting the synthesis of trichothecenes.^22^ The use of KS fertilization in wheat crops to improve the defense mechanism against fungal contamination is uncommon.

### 3.2 Performance of Methods of Determination of TCTBs

The chromatogram of the TCTBs is in the Supporting Information. The order of mycotoxin elution in the chromatographic system was DON-3G (4.8 min), NIV (5.1 min), DON (5.7 min), 15-ADON (7.2 min), and 3-ADON (7.6 min). Its good resolution (0.96 to 4.2) allowed quick separation, identification, and quantify trichothecenes. Similar chromatographic performance was found by Cerqueira et al.^27^ and Borba et al.^21^ who used HPLC-PAD for separating TCTB in oats and wheat products, respectively.

The performance parameters of the validated method for determination in wheat samples are given in Table 1. The found indicators were aligned with the guidelines by ANVISA^34^ and EC.^35^

**Table 1:** Performance of the Analytical Method for TCTBs^a^

parameters	DON-3G	NIV	DON	15-ADON	3-ADON
	chromatographic parameters—HPLC-PAD				
Tr (min)	4.8	5.1	5.7	7.2	7.6
Rs	-	0.9	1.6	4.2	0,96
solvent curve (ug/mL)	*y* = 24642*x* + 300	*y* = 52641*x* + 593	*y* = 23387*x* + 1517	*y* = 58388*x* + 2677	*y* = 30294*x* + 1647
linearity (ug/mL)	0.015–2.5	0.015–2.5	0.025–2.5	0.015–3.0	0.015–2.80
LODinst. (ug/mL)	0.015	0.013	0.025	0.015	0.018
LOQ inst. (ug/mL)	0.043	0.052	0.069	0.060	0.060
	validation parameters of the QuEChERS method				
recovery (%) *	93.0	92.3	88.0	94.0	90.2
RSD (%)	16.7	15.1	16.4	14.2	15.8
LODm (μg/kg)	18.5	19.2	18.4	19.0	23.3
LOQm (μg/kg)	55.5	57.6	93.56	67.3	78.4
linearity (μg/kg)	50.0–1900	46.0–1700	63.6–2500	60.7–2500	78.2–2500
matrix curve (ug/mL)	*y* = 29658*x* + 392	*y* = 54742*x* – 367	*y* = 23237*x* + 285	*y* = 33177*x* + 562	*y* = 43716*x* + 1619
*R* ^2^	0.9969	0.9947	0.9948	0.9968	0.9980

a Tr: retention time, Rs: resolution, LODinst: limit of detection of the instrument, LOQ: limit of quantification of the instrument; % recovery*: media recovery of three levels (1 × LOD, 3 × LOD, 5 × LOD); LODm: limit of detection of the method, LOQm: limit of quantification of the method.

4

The standard curve of each TCTB showed linearity ranging from 0.015 to 3.0 μg/mL and media correlation coefficient 0.98. The detection limit of the instrument (LODi) was the concentration of each standard solution that generated a signal 3-fold higher than the noise (Table 1). To infer the quantification limits of the TCTBs method (LOQm: 55.5 to 78.4 μg/kg), the LODi and the dilutions in the sample preparation were considered. The interferences in the extract were estimated from matrix standard curves, the mean values of which were below 20% for all TCTBs. The TCTBs matrix curves, which had similar linearity (from 50 to 2500) and correlation coefficient (0.99), were used to quantify the contaminants in the samples. Mean recoveries at 3 levels of contamination of each TCTB were 93% for DON-3G, 92.3% for NIV, 88% for DON, 94 for 15-ADON, and 90.2% for 3-ADON. The standard deviations (RSD) were between 14.2% and 16.7%.

### 4.1 Profile and Sum of Trichothecenes in Wheat Samples

Co-contamination with TCTB was common in the study and showed that the FGSC had diverse chemotypes in the region, a fact that indicated that DON should not be the only concern. Table 2 shows the summary of TCTBs contamination as responses to agronomic variables.

**Table 2:** Summary of Contamination with TCTBs in Wheat samples^a^

summary	number and type of TCTB
total samples	48
TCTBs frequency (number of contaminated samples)	DON (46)> 15-ADON (43)>NIV (31)> D3G (30)>3-ADON (5)
sum of TCTB > 1000 μg/kg	22 samples
highest level of TCTB (μg/kg)	15 ADON (1640), D3G (1224)
	DON (1203), NIV (604), 3-ADON (354)
samples contaminated with 1 TCTB	10
Co-contamination (sample number)	2 TCTB = 20
	3 TCTB = 13
	4 TCTB = 5
	5 TCTB = 0
DON contamination > LOQ (contaminated samples per year)	2021 = 12
	2022 = 16
	2023 = 2
15-ADON contamination > LOQ (contaminated samples per year)	2021 = 16
	2022 = 14
	2023 = 10
NIV contamination > LOQ (contaminated samples per year)	2021 = 0
	2022 = 4
	2023 = 14
DON- 3G contamination > LOQ (contaminated samples per year)	2021 = 5
	2022 = 13
	2023 = 0
3-ADON contamination > LOQ (contaminated samples per year)	2021 = 2
	2022 = 0
	2023 = 0

a TCTBs: group B trichothecenes; LOQ: limit of quantification.

The frequency of detection of TCTBs in three years of experiments was DON (95.8%), 15-ADON (89.6%), NIV (64.6%), DON-3G (62.5%), and 3-ADON (10.4%). Annual frequencies showed that contamination levels were higher in 2021 than in other years. Values of contaminants above the LOQ were found in 65.2%, 93.0%, 58.1%, 63.3%, and 40% for DON, 15-ADON, NIV, DON-3G, and 3-ADON, respectively.

The 15-ADON contamination level ranged from 135 to 1640 μg/kg. It was the most frequent and the one that had the highest detected level of TCTBs, followed by DON-3G (141 to 1224 μg/kg). Other authors have reported that in subtropical regions, 15-ADON was frequently found.^4,33^ Considering the crop year and the sum of the contaminants, the decreasing order of toxicological potential of the samples was 2021 = 2022 > 2023 that reinforced the importance of climate parameters for production of total TCTB in wheat.^2,8^

In 2021, in preanthesis, anthesis, and postanthesis, the mean temperature was higher (27.7 °C) than in 2022 (23.4 °C) and in 2023 (26.3 °C) combined with lower rainfall and relative humidity (75.3%) (Supporting Information). The most contaminated sample was the susceptible cultivar harvested in 2022 in the irrigated control field.

This study showed that co-contamination with TCTBs was common in all agronomic conditions and, considering that there is interconversion among chemical forms to DON, it indicated that DON determination alone may not represent the actual exposure in the wheat supply chain.^7,8,36^

The highest DON MTL proposed for wheat grains is 2000 μg/kg,^2^ which was not found for DON in this study. However, the sum of TCTBs in two samples would be rejected because they exhibited contamination above this MTL. The sum of TCTBs of 20 samples was above the MTL recommended for wheat flour by some countries’ legislations since it is 1000, 700, 500 μg/kg and lower.^8,33,34^

In the study, ranges of these recommended MTL were considered to classify samples into four groups, according to their sums of TCTBs (μg/kg) and to infer about the toxigenic potential of each group. It was range 1: from 2967 to 1040 μg/kg (>1000 μg/kg); range 2: from 939 to 718 μg/kg (>700 μg/kg); range 3: from 697 to 546 μg/kg (>500 μg/kg); and range 4: from 483 to 190 μg/kg (<500 μg/kg). In this criterion, the order of potential toxicity is decreasing.

Sum concentrations above 1000 μg/kg were found in 41.7% of contaminated samples; 65% was grown in 2021 and DON, and 15-ADON were the predominant TCTBs. Other reports highlighted that high temperature in the preanthesis stage of wheat plants favored 15-ADON production.^2,10,25^ It occurred in samples of 2021, when the rainfall was low, the maximum temperature was 33 °C, and the mean relative humidity was 67.7% in the anthesis stage, where behavior is aligned with the literature reports.^4,8,10,25^

Table 3 shows the summary of relations between the sum of TCTBs and the agronomic variables (crop year, seed cultivar, irrigation system, and treatments T1, T2, T3, and T4).

**Table 3:** Variables and Sums of TCTBs in Wheat^a^ ^,^ ^b^

variables/TCTB sum	range 1	range 2	range 3	range 4
sample number (48)	20	10	9	9
year 2021	13	1	2	0
year 2022	5	6	2	3
year 2023	2	3	5	6
susceptible cultivar	12	4	6	6
MR cultivar	8	6	3	3
irrigated field	10	5	3	6
rainfed field	10	5	6	3
fungicide (T1)	4	2	5	1
fungicide + KS (T2)	5	4	2	3
KS (T3)	3	2	3	4
control (T4)	8	2	0	2

a TCTBs: group B trichothecenes; MR: moderately resistant; KS: potassium silicate.

b Range 1:2967–1040 μg/kg (>1000 μg/kg); range 2:939–718 μg/kg (>700 μg/kg); range 3:697–546 μg/kg (>500 μg/kg); range 4:483–190 μg/kg (<500 μg/kg).

The susceptible cultivar had 52.2% of the total contaminated samples, as expected. It showed that MR cultivars, in any environmental condition and agronomic management, showed the trend of preventing mycotoxicological contamination.^23,37^ The effect of the irrigation system did not show any clear trend on the co-contamination profile. Higher levels than the LOQ occurred in 23% of samples in control experiments, a fact that may represent random effects of variables in the field.

Fungicides that belong to mesosystemic and systemic classes of chemical groups Strobilurins and Triazole have been recommended to prevent *Fusarium ssp* contamination in many wheat-producing regions.^16,36,38^ The sum of TCTBs above 1000 μg/kg was found in 29 samples (60.4%); 6 of them were treated with fungicides. It represented 50% of samples submitted to fungicide treatment. By comparison to control samples, when fungicide was applied, there was a trend to reduce the level of contamination. Although the scarce evaluation of fungicides effects on TCTBs co-contamination, it was expected a positive effect on DON derivates prevention. Only 4 of 12 experiments had contamination below the LOQ with this treatment. Scaglioni et al.^22^ showed that some chemical fungicides were able to avoid fungal diseases in cereal crops but have low capacity to prevent TCTBs synthesis in wheat.

Association of fungicides and KS avoided contamination above 1000 μg/kg in 58.3% (7/12) of treated field, suggesting that the treatment should have the potential to reduce contamination with TCTBs. Application of KS alone prevented contamination above 1000 μg/kg in 66.7% of samples submitted to the treatment. Regarding DON, the KS treatment showed the lowest value (122 μg/kg) in a sample from a susceptible cultivar grown in a rainfed system in 2021. Regarding the sum of TCTBs (1343 μg/kg), the preventive potential of KS was not clear, despite it showing a decreasing trend. There are reports of the beneficial effects of KS application to wheat cultures and other plants.^39–42^ Thus, it reinforced its recommendation as an alternative to prevent environmental damage caused by fungicide.^8^ While KS has previously been used for boosting resistance to climate and salt stress, this study is the first to report its foliar application to wheat against fungal species and toxin production. The pioneering results suggest that application of KS, which seems to improve the defense mechanism of plants, should be deeply evaluated because it may be adopted as a friendly and cost-effective tool to reduce application of fungicides and prevent TCTB in grains.

### 4.2 Agronomic Variables and Co-contamination with TCTB

To correlate agronomic variables and TCTB levels, the value which was lower than the LOQ was used as the LOQm values, and results expressed as “ND” (not detected) were excluded from the statistical analysis (Table 4). The statistical analysis confirmed the significance of some previously discussed behavior.

**Table 4:** Analysis of Variance and the Fischer Test of Effects of Sources of Variation on Concentrations of Mycotoxins Deoxynivalenol (DON), Nivalenol (NIV), 3-Acetyl-DON (3ADON), 15-Acetyl-DON (15ADON), 3-Acetyl-DON (D3G), and Their Sum^a^

factor	source of variation	DON		NIV		3ADON		15ADON		D3G		SUM	
		*T*	*N* (μg/kg)	*T*	*N* (μg/kg)	*T*	*N* (μg/kg)	*T*	*N* (μg/kg)	*T*	*N* (μg/kg)	*T*	*N* (μg/kg)
year	2021	5.3^b^	212	Nd	Nd	5,6^a^	270	6.8^a^	979	5.2^a^	259	7.2^a^	1424
	2022	5.6^a^	371	4.8^b^	120	Nd	Nd	5.5^c^	361	5.4^a^	236	6.9^b^	1089
	2023	4.8^c^	128	5.6^a^	295	5.0^b^	108	6.0^b^	497	Nd	Nd	6.5^c^	766
	*p*-value (F)	<0.001		<0.001		0.037		<0.001		0.405		<0.001	
	standard error	0.09		0.10		0.23		0.13		0.12		0.07	
cultivation	irrigated	5.3^a^	281	5.1^a^	177	4.7^a^	108	6.2^a^	682	5.3^a^	253	6.9^a^	1137
	dryland	5.1^a^	191	5.2^a^	230	5.3^a^	216	6.1^a^	576	5.3^a^	240	6.9^a^	1048
	p-value (F)	0.225		0.449		0.284		0.696		0.874		0.873	
	standard error	0.09		0.10		0.23		0.13		0.12		0.07	
grain variety	moderately resistant	5.2^a^	221	5.1^a^	177	4.7^a^	108	6.1^a^	567	5.2^a^	241	7.0^a^	1029
	susceptible	5.2^a^	254	5.3^a^	231	5.3^a^	216	6.1^a^	699	5.4^a^	252	7.0^a^	1156
	*p*-value (F)	0.892		0.369		0.284		0.941		0.385		0.912	
	standard error	0.09		0.10		0.23		0.13		0.12		0.07	
treatment	fungicide + KS	5.1^b^	187	5.3^a^	237	Nd	Nd	6.1^ab^	572	5.5^a^	305	6.8^ab^	999
	fungicide	5.2^b^	230	5.1^a^	193	5.9^a^	354	6.0^ab^	562	5.3^a^	225	6.8^ab^	1021
	KS	5.0^b^	164	5.1^a^	206	4.9^b^	134	5.9^b^	487	5.1^a^	259	6.7^b^	941
	control	5.6^a^	376	5.1^a^	181	4.7^b^	108	6.5^a^	876	5.2^a^	198	7.1^a^	1410
	*p*-value (F)	0.126		0.798		0.178		0.289		0.596		0.220	
	standard error	0.09		0.10		0.23		0.13		0.12		0.07	

a Different letters in each column, for each factor, indicate statistically distinct means by the Fischer test (*p* < 0.10). *T* = result transformed by *T* = ln (*x* + 1). *N* = real result without any transformation. Nd = not detected.

The crop year was confirmed as an important factor in the TCTBs profile and their sums. The exception was DON-3G, which exhibited equal levels in 2021 and 2022 and was not detected in 2023. The decreasing order of contamination with TCTBs in relation to crop/year for DON was 2022 > 2021 > 2023; for NIV: 2023 > 2022, not detected in 2021; for 3-ADON: 2021 > 2023 and not detected in 2022; and for 15-ADON: 2021 > 2023 > 2022. Samples collected in 2021, when high temperature and low rainfall occurred in the stages of anthesis and grain filling, showed higher synthesis of TCTBs by the FGSC than crops harvested in 2022 and 2023.

Climate variables may cause changes in the DON pathway and may explain why 15-ADON and DON-3G levels were higher than the ones of the other crops under study. The decreasing order of the sum of contaminants was 2021 > 2022 > 2023, a behavior that is aligned with each level of individual mycotoxin. A similar effect was reported by Duffeck et al.^1^ who evaluated 461 isolates of *F. graminearum* from wheat, barley, and rye collected in regions with different climate conditions. Isolates from samples collected in regions with higher temperatures showed a prevalence of the 15-ADON chemotype.

The irrigation system and cultivar susceptibility did not show any significant effects on the individual levels or sums of TCTBs. This finding should be important to the establishment of strategies to protect crops against the expected drastic environmental conditions. It also suggested that the MR cultivar requires further improvement against contamination with the FGSC.

In the samples from the control treatment DON, 15-ADON, and DON-3G levels and frequency stood out. Application of fungicide KS and fungicide + KS did not have any significant effect on NIV and DON-3G levels. The same treatments showed an intermediate effect on the decrease in the sum of TCTBs. Samples treated with KS had lower contaminant levels than control samples, a fact that means a promising alternative for improving protection against TCTBs, that should be deeply studied before application.

3-ADON was not detected in the treatment with fungicide + KS, while the treatment with fungicide exhibited samples with their low frequency of contamination, and it may be a random behavior because they did not have any significant difference.

Kléber et al.^43^ evaluated the efficacy of fungicides to control DON, 15A-DON, and DON-3G during four wheat crops in France. They used commercial fungicides based on prothioconazole + tebuconazole, prothioconazole + fluoxastrobin, and prothioconazole + trifloxystrobin. There were no significant differences, despite the reduction in DON concentrations and other mycotoxins by comparison to the negative control. Getahun et al.^44^ evaluated both fungicides tebuconazol and propiconazole in wheat crops in Ethiopia. DON contamination was reduced by comparison to the MR cultivar control. Triazole fungicides showed high efficacy to mitigate Giberela and DON while Strobilurins, in general, was not efficient.^35^

Although combinations of fungicide types have improved efficacy against mycotoxicological contamination, as other studies also reported,^8,44,45^ they did not stand out as a great solution to reduce the level of TCTB. Although they were able to avoid fungal diseases, the effect on the TCTBs profile remains a concern.

Fertilization with KS significantly reduced the TCTBs levels. This approach, which induces the natural defense mechanisms of hosts and its uses in conjunction with fungicides, demonstrated potential for improving protection efficiency while remaining cost-effective.

Frequency and the level of co-contamination with TCTBs in wheat, mainly 15-ADON, impacted the sum of DON derivatives. It reinforces that the establishment of MTL for DON alone does not reflect actual health protection since derivate forms may interconvert to DON in the food chain or in consumers’ digestive systems (Lemos et al., 2025). It should be highlighted that TCTBs cytotoxicity decreasing order is NIV >15-ADON ≈ DON >3 ADON ≫ D3G.^46^ The frequent co-contamination is a concern.

In summary, crop management influenced FGSC production of chemical DON forms in wheat grain, as shown by consistent co-contamination in the study. DON (95.8%), 15-ADON (89.6%), NIV (64.6%), DON-3G (62.5%), and 3-ADON (10.4%) were found in the wheat samples simultaneously. Two samples had TCTBs levels exceeding the MTL (2000 μg/kg) recommended for crude grain. Twenty samples (42%) would be unsuitable for human consumption, if the MTL considered was 1000 μg/kg.^34^ If only DON was considered, then one sample would be unsuitable.

The expected effect of climatic variables on the TCTBs production profile was reinforced with regard to its importance for frequency and levels of co-occurrence. High temperature (33 °C) and low rainfall (67 MRU %) were aligned with 15-ADON and DON-3G levels above 1000 μg/kg.

Strobilurins and Triazole fungicides, together with KS fertilization and KS fertilization alone, were expected to reduce the level of TCTBs and their sum in wheat samples, but further studies are needed to recommend them as preventing tools. It is advisable to review the current MTL for DON and its derivatives and prevention protocols to ensure wheat products are safe.

## Supplementary Material



## Notes

Chromatograms of TCTBs and climatic variables in the years of the experiments (PDF)

## References

[1] Duffeck M. R., Del Ponte E. M., Esker P. D. (2021). Multifaceted Insights on Fusarium
Head Blight in Small Grains in Pennsylvania. Plant Health Prog..

[2] Del
Ponte E. M., Moreira G. M., Ward T. J., O’Donnell K., Nicolli C. P., Machado F. J., Duffeck M. R., Alves K. S., Tessmann D. J., Waalwijk C. (2022). Fusarium graminearum
Species Complex: A Bibliographic Analysis and Web-Accessible Database
for Global Mapping of Species and Trichothecene Toxin Chemotypes. Phytopathol.

[3] ABIA Associação Brasileira da Indústria de Alimentos [Internet]. 2025 [accessed December 2, 2025]. Available in https://www.abia.org.br/.

[4] Junior, W. C. J. ; Badiale-Furlong, E. ; Agapto, J. P. ; Oliveira, D. N. ; Afférri, F. S. ; Carmassi, A. L. ; Regional Variability in Mycotoxin Contamination of Brazilian Wheat: Implications for Food Security and Management Strategies. In Triticum - The Pillar of Global Food Security; IntechOpen, 2025.

[5] Antonissen G., Martel A., Pasmans F., Ducatelle R., Verbrugghe E., Vandenbroucke V. (2014). The Impact of Fusarium
Mycotoxins on Human and Animal Host Susceptibility to Infectious Diseases. Toxins (Basel)..

[6] Alisaac E., Mahlein A. K. (2023). Fusarium Head Blight on Wheat: Biology, Modern Detection
and Diagnosis and Integrated Disease Management. Toxins.

[7] Lemos A. C., Borba V. S., Scaglioni P. T., Badiale-Furlong E. (2025). Assessment
of group B trichothecene contamination in bread: Correlation during
breadmaking and simulation of gastrointestinal digestion. Food Chem..

[8] Leslie J. F., Moretti A., Mesterházy A.
´., Ameye M., Audenaert K., Singh P. K. (2021). Key Global Actions for
Mycotoxin Management in Wheat and Other Small Grains. Toxins.

[9] Pereira C. B., Ward T. J., Del Ponte E. M., Mara Moreira G., Busman M., McCormick S. P., Feksa H. R., De Almeida J. L., Tessmann D. J. (2021). Five-year survey
uncovers extensive diversity and temporal
fluctuations among fusarium head blight pathogens of wheat and barley
in Brazil. Plant Pathol..

[10] Lee H. J., Ryu D. (2017). Worldwide Occurrence of Mycotoxins in Cereals and Cereal-Derived
Food Products: Public Health Perspectives of Their Co-occurrence. J. Agric. Food Chem..

[11] Haile J. K., N’Diaye A., Walkowiak S., Nilsen K. T., Clarke J. M., Kutcher H. R. (2019). Fusarium
Head Blight in Durum Wheat: Recent
Status, Breeding Directions, and Future Research Prospects. Phytopathology.

[12] Ascari J. P., Barro J. P., Santana F. M., Padua J. M. V., Maciel J. L. N., Lau D. (2021). Sequential Post-Heading
Applications for Controlling
Wheat Blast: A 9-Year Summary of Fungicide Performance in Brazil. Plant Dis..

[13] Juroszek P., Racca P., Link S., Farhumand J., Kleinhenz B. (2020). Overview on the review articles published during the
past 30 years relating to the potential climate change effects on
plant pathogens and crop disease risks. Plant
Pathol..

[14] Krnjaja V., Mandić V., Lević J., Stanković S., Petrović T., Vasić T. (2015). Influence of N-fertilization
on *Fusarium* head blight and mycotoxin levels in winter
wheat. Crop Prot..

[15] Yoshida M., Nakajima T., Tonooka T. (2008). Effect of nitrogen
application at
anthesis on Fusarium head blight and mycotoxin accumulation in breadmaking
wheat in the western part of Japan. J. Gen.
Plant Pathol..

[16] Dos
Santos I. D., Pizzutti I. R., Dias J. V., Fontana M. E. Z., Souza D. M., Cardoso C. D. (2021). Mycotoxins in wheat flour: occurrence
and co-occurrence assessment in samples from Southern Brazil. Food Addit Contam Part B-Surveill.

[17] Paul P. A., Bradley C. A., Madden L. V., Dalla Lana F., Bergstrom G. C., Dill-Macky R. (2018). Effects of Pre- and
Postanthesis Applications of Demethylation Inhibitor Fungicides on
Fusarium Head Blight and Deoxynivalenol in Spring and Winter Wheat. Plant Dis..

[18] Machado F. J., Santana F. M., Lau D., Del Ponte E. M. (2017). Quantitative
Review of the Effects of Triazole and Benzimidazole Fungicides on
Fusarium Head Blight and Wheat Yield in Brazil. Plant Dis..

[19] Marques L. N., Pizzutti I. R., Balardin R. S., Dos Santos I., Dias J. V., Stefanello M. T., Serafini P. (2017). Occurrence of mycotoxins
in wheat grains exposed to fungicides on fusarium head blight control
in southern Brazil. J. Environ. Sci. Health
B.

[20] West J. S., Holdgate S., Townsend J. A., Edwards S. G., Jennings P., Fitt B. D. L. (2012). Impacts of changing
climate and agronomic factors on
fusarium ear blight of wheat in the UK. Fungal
Ecol..

[21] Borba V. S., Lemos A. C., Rodrigues M. H. P., Cerqueira M. B. R., Badiale-Furlong E. (2023). Type B trichothecenes
in cakes and
their interaction with matrix components. Food
Control.

[22] Scaglioni P. T., Scarpino V., Marinaccio F., Vanara F., Furlong E. B., Blandino M. (2019). Impact of microalgal
phenolic extracts on the control
of Fusarium graminearum and deoxynivalenol contamination in wheat. World Mycotoxin J..

[23] Edwards S. G., Jennings P. (2018). Impact of agronomic factors on fusarium mycotoxins
in harvested wheat. Food Addit. Contam.,:Part
A.

[24] Menke J., Weber J., Broz K., Kistler H. C. (2013). Cellular Development
Associated with Induced Mycotoxin Synthesis in the Filamentous Fungus
Fusarium graminearum. PLoS One.

[25] Rossi, M. . Mapa PedolóGico Do Estado De SãO Paulo: Revisado E Ampliado; Memórias do Instituto Florestal. 2017.

[26] Zadoks J. C., Chang T. T., Konzak C. F. (1974). A decimal
code for the growth stages
of cereals. Weed Res..

[27] Cerqueira M. B. R., de Borba V. S., Rodrigues M. H. P., Silveira C. O., Badiale-Furlong E., Kupski L. (2023). Reliable and Accessible
Method for Trichothecenes Type
B Determination in Oat Products. Food Anal.
Methods.

[28] Foroud N. A., Baines D., Gagkaeva T. Y., Thakor N., Badea A., Steiner B. (2019). Trichothecenes
in Cereal Grains – An Update. Toxins.

[29] Hohn, T. M. , McCormick, S. P. , Alexander, N. J. , Desjardins, A. E. , Proctor, R. H. Function and Biosynthesis of Trichothecenes Produced by Fusarium Species. Molecular Genetics of Host-Specific Toxins in Plant Disease, Proceedings of the 3rd Tottori International Symposium on Host-Specific Toxins, August 24–29; Em: Kohmoto K, Yoder OC , Eds. oganizadores.; Springer, Daisen, Tottori, Japan, 1998, [cited December 2, 2025].

[30] Schöneberg T., Jenny E., Wettstein F. E., Bucheli T. D., Mascher F., Bertossa M. (2018). Occurrence of *Fusarium* species
and mycotoxins in Swiss oats—Impact of cropping factors. Eur. J. Agron..

[31] Lavra
Vieira D., de Oliveira Barbosa V., Oliveira de Souza W. C., Gonçalves da Silva J., Malaquias J. B., de Luna Batista J. (2015). Potassium silicate-induced resistance against blackfly
in seedlings of Citrus reticulata. Fruits.

[32] Wegulo S. N., Bockus W. W., Nopsa J. H., De Wolf E. D., Eskridge K. M., Peiris K. H. S. (2011). Effects of Integrating Cultivar Resistance
and Fungicide Application on Fusarium Head Blight and Deoxynivalenol
in Winter Wheat. Plant Dis..

[33] Dos
Santos G. B., de Oliveira Coelho M.
A., Del Ponte E. M. (2022). Incidence-severity
relationships in non-treated and fungicide-treated wheat head blast
epidemics in Brazil. Eur. J. Plant Pathol..

[34] ANVISA. AN de VS Res^o^luçÃOão Da Diretoria Colegiada - RDC no 166, De 24 De Julho De, 2017.

[35] European Comission, EC. EUR-lex . 2024 [accessed December 2, 2025. Available: https://eur-lex.europa.eu/PT/legal-content/summary/methods-of-sampling-and-analysis-for-the-control-of-levels-of-certain-contaminants-in-foodstuffs.html.

[36] Knutsen H. K., Alexander J., Barregård L., Bignami M., Brüschweiler B., Ceccatelli S., Cottrill B., Dinovi M., Grasl-Kraupp B. (2017). Risks to human and animal health related to the presence of deoxynivalenol
and its acetylated and modified forms in food and feed. EFSA J..

[37] Gaire R., Sneller C., Brown-Guedira G., Van Sanford D., Mohammadi M., Kolb F. L. (2022). Genetic
Trends in Fusarium
Head Blight Resistance from 20 Years of Winter Wheat Breeding and
Cooperative Testing in the Northern U.S.A. Plant
Dis..

[38] Pirgozliev S. R., Edwards S. G., Hare M. C., Jenkinson P. (2002). Effect of
Dose Rate of Azoxystrobin and Metconazole on the Development of Fusarium
Head Blight and the Accumulation of Deoxynivalenol (DON) in Wheat
Grain. Eur. J. Plant Pathol..

[39] Saudy H. S., Salem E. M. M., Abd El-Momen W. R. (2023). Effect
of Potassium Silicate and
Irrigation on Grain Nutrient Uptake and Water Use Efficiency of Wheat
Under Calcareous Soils. Gesunde Pflanz..

[40] Aurangzaib M., Ahmad Z., Jalil M. I., Nawaz F., Shaheen M. R., Ahmad M., Hussain A., Ejaz M. K., Tabassum M. A. (2022). Foliar
Spray of Silicon Confers Drought Tolerance in Wheat (Triticum aestivum
L.) by Enhancing Morpho-Physiological and Antioxidant Potential. Silicon.

[41] Feghhenabi F., Hadi H., Khodaverdiloo H., Van Genuchten M. T., Pessarakli M. (2022). Improving wheat (Triticum aestivum
L.) antioxidative
defense mechanisms against salinity stress by exogenous application
of potassium silicate. J. Plant Nutr..

[42] Li W., Li M., Xu Y., Shi Y. (2021). Effects of Potassium Silicate Fertilizer
on Photosynthetic Characteristics and Yield in Winter Wheat (Triticum
Aestivum L.). Bangladesh J. Bot..

[43] Kleber A., Gruber-Dorninger C., Platzer A., Payet C., Novak B. (2023). Effect of
Fungicide Treatment on Multi-Mycotoxin Occurrence in French Wheat
during a 4-Year Period. Toxins.

[44] Getahun M., Fininsa C., Mohammed A., Bekeko Z. (2024). Integrated management
of wheat (Triticum aestivum L.) Fusarium head blight and deoxynivalenol
contamination through host resistance and fungicide application in
Ethiopia. J. Crop Sci. Biotechnol..

[45] Liu J., Jiang J., Guo X., Qian L., Xu J., Che Z. (2022). Sensitivity
and Resistance Risk Assessment of Fusarium
graminearum from Wheat to Prothioconazole. Plant
Dis..

[46] Lemos A. C., Borba V. S., Badiale-Furlong E. (2021). The impact of wheat-based food processing
on the level of trichothecenes and their modified forms. Trends Food Sci. Technol..

