# Supplementary material for: Impact of Wheat Crop Management on Co-occurrence of Group B Trichothecenes: Crop Practices Influencing DON and Derivatives in Wheat
Source: J Agric Food Chem. 2026 Mar 26;74(13):11150–7. doi: 10.1021/acs.jafc.6c00387 (PMC13067350; doi:10.1021/acs.jafc.6c00387)
Supplement: Supplementary file 1 [file jf6c00387_si_001.pdf]

Title: Impact of Wheat Crop Management on Co-Occurrence of Group B Trichothecenes

Running Title: Crop Practices Influencing DON and Derivatives in Wheat

Agápto, João Paulo[a]; de Jesus Júnior, Waldir Cintra[a]; Afférri, Flávio Sérgio[a]; Carmassi, Alberto Luciano[a]; Andressa Cunha Lemos[b]; Badiale-Furlong, Eliana[b*]; Scaglioni, Priscila Tessmer[b]*

[a]Universidade Federal de São Carlos, Campus Lagoa do Sino, Centro de Ciências da Natureza - Buri, São Paulo State- Brazil 18290-000

[b]Universidade Federal do Rio Grande, Campus Carreiros, Escola de Química e Alimentos- Rio Grande, Rio Grande do Sul State – Brazil – 96203-900

*Corresponding authors: dqmebf@furg.br and priscilascaglioni@gmail.com

1.Supplementary Material

Figure 1. Chromatograms of TCTBs

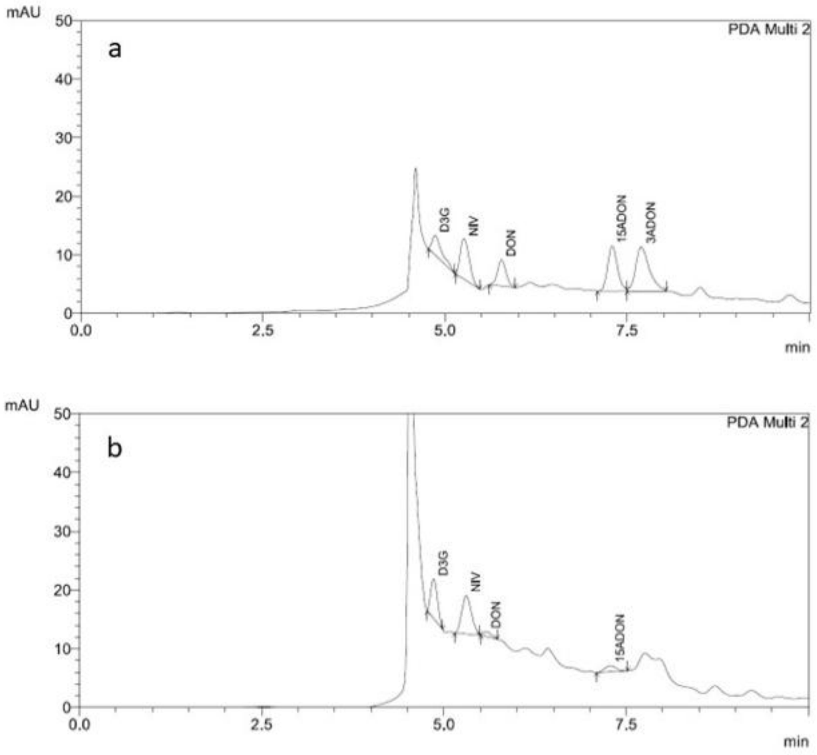

a. Matrix curve of TCBs
b. Sample chromatogram

2. Supplementary Material

Climatic variables in the years of the experiments

Years	2021			2022			2023		
Stages	PAT	AT	PsAT	PAT	AT	PsAT	PAT	AT	PsAT
R mm			16.5	13	29.5	23		2.5	2.5
Max TºC	24.84	33.16	24.96	25.78	24.38	20.15	24.9	28.45	25.54
Min TºC	11.3	10.18	13.97	11.51	10.69	10.99	11.23	9.8	13.68
Mean TºC	17.9	21.7	19.5	18.6	17.5	15.6	18.1	19.1	19.6
MRU %	79.8	67.3	78.5	82.6	82.0	86.8	83.2	79.6	85.12

PAT: pre-anthesis; AT: anthesis; PsAT: post-anthesis; R: rainfall; MRU: mean relative humidity; TºC: temperature as centigrade; Max: maximum; Min: minimum.
